# Case of Amnesia, or Loss of Language

**Published:** 1829-07-01

**Authors:** 


CASE OF AMNESIA ; OR, LOSS OF LANGUAGE. By S. JACKSON, M.D. Assistant Professor of the Institutes &c. of Clinical Medicine in the University of Pennsylvania. With additional Cases and Remarks.

A failure of the memory, general or partial, is extremely common, though little noticed. Those who may take the trouble to make enquiries, will find that very many patients acknowledge the defect in question, though few of them state it in the catalogue of their ailments, unless cathechised on that particular point. Partial losses, such as of proper names, nouns-substantive, or certain languages, arrest the attention in a peculiar manner, and afford data for arguments in favour of and against phrenology. At present we shall not enter on argumentation, but only state facts. The first which we shall put upon record in our pages, on the present occasion, is the following from the American Journal of the Medical Sciences, No. VI. February 1829.

" The Rev. Mr. R. the subject of this case, is aged forty-eight years ; he is of the sanguine temperament, ruddy complexion, light-coloured hair and eyes, and has lately manifested a strong tendency to obesity; his health for many years has been excellent; he is not subject to head-ache, or to any nervous symptoms. His intellectual faculties are of a high order, but have not been as actively employed as formerly, and he has experienced some mental anxiety: his temper is placid, with a disposition bordering on gaiety.

" On the 5th of September last, early in the morning, he awoke with headache, after a restless night. He had, the preceding evening, been exposed to the night air, which had lowered in temperature, and perspiration, which was usually copious, received a sudden check. He took some castor oil, which acted freely in a short time, after which he again laid down. About eleven o'clock, the Rev. Mr. H. who resides in the same dwelling, went into his room to inquire respecting his health, and was surprised to find Mr. R. could not answer his questions. Alarmed at this circumstance, he immediately requested me to visit Mr. R.

" I found my patient in bed, evidently in the full possession of his senses, but incapable of uttering a word. I examined the tongue, and ascertained it was not paralysed, but could be moved in every direction. All my questions were perfectly comprehended, and answered by signs; and it could be plainly seen, by the smile on the countenance, after many ineffectual attempts to express his ideas, that he was himself surprised and somewhat amused at his peculiar situation.

"The face at this time was flushed, the pulse full and somewhat slow, and to my inquiries if he suffered pain in the head, he pointed to the front of his forehead as its seat.

"I directed hot water to be brought in a bucket, for a pediluvium, and made preparations to draw blood. Mr. R. exhibited at this time a strong desire to speak, and, after a great many ineffectual efforts, endeavoured to make me comprehend his meaning by signs. Finding I could not understand him, he made a sign that he would write. When furnished with pen and paper, he attempted to convey his meaning, but I saw he could not recall words, and that he had written an unintelligible phrase: it was 'Didoes doe the doe.'

"Forty ounces of blood were drawn from the arm, and before the operation was completed, speech was restored, though a difficulty continued as to the names of things, which could not be recalled. The bleeding and pediluvium produced some faintness, and he was placed in bed.

"The loss of speech appearing to recur again, in fifteen minutes, ten ounces more of blood were abstracted, and sinapisms applied to the arms, legs, and thighs, alternately: the skin became moist and the head-ache was relieved.

"Mr. R. now communicated to me, that when he made the attempt to write, he had intended to inform me he had already used a foot bath, and I might see the floor still wet, where the water had been spilt.

"The sleep that night was disturbed by uneasiness and throbbing in the head, which disappeared in the course of the 6th, and no further return of the affection has occurred.

"In an analysis of this case, we are presented with the following facts: 1st. Sudden suppression of the cutaneous transpiration, succeeded by cerebral irritation and determination of blood to the brain; 2d. frontal pain immediately over the eyes; 3d. perfect integrity of the sensations and voluntary movements; 4th. the general operations of the intellect undisturbed; ideas formed, combined and compared; those of things, of events, of time, recalled without difficulty; 5th. loss of language, or of the faculty of conveying ideas by words, though not by signs: this defect was not confined to spoken language, but also extended to written language.

"The inferences to be drawn from these facts, are, 1st, that as the cerebral irritation produced no general affection or disturbance of the functions of the brain, it was local or limited; and 2d, as loss of language was the only functional derangement of the intellectual faculties, that faculty must have been connected with the portion of the brain, the seat of the irritation; and 3d, that an organ of language exists in the brain. This case lends a strong confirmation to the general truth of the doctrines of Phrenology."

In a late number of the *Archives Generales* we observe the case of a gentleman, aged 56, who, while playing at the game called *tric-trac* in a warm room, felt a sudden pain in his left temple, and, at the same moment, lost the power of expressing his thoughts in appropriate language. His memory betrayed him every time he attempted to use a substantive word—all words of that kind being re-placed by *sonnez* and *six-cinque*, terms employed in the game he had been playing. When the physician (Dr. Chailly) saw him, his face was flushed, and he indicated by gestures that he had pain in the left temple. By copious bleeding, general and local, and revulsive applications, the patient was entirely restored.

In Hufeland's Journal for November, 1828, we observe the case of a physician, Dr. Schoerf, author of several estimable works, who became affected with a severe fever, and was in much danger of life. Nevertheless he recovered from the fever; but found himself completely destitute of all knowledge of the Latin language, in which he had been a proficient. In all other respects his memory was as perfect as ever. He was quite unable to write his prescriptions in Latin, and was greatly distressed on this account. Gradually, however, the memory of the Latin language returned, and that in proportion as his physical strength was restored.

The editor of a French journal (in which this case is noticed) sarcastically asks—"how will the phrenologists explain this curious fact? Is there an organ for the MEMORY of *Latin*?"—Without being deeply versed in phrenology, we believe we may aver that phrenologists have never appropriated any organ for *memory*. But granting there was an organ of *language*—is there any thing very absurd in supposing that the function of this organ may be *partially* disordered? The editor abovementioned would not deny that the brain is the organ of thought—and yet, in a case of monomania, (not an extremely rare occurrence,) he would be somewhat puzzled to account for the disorder of intellect, excepting on the supposition that a function may be *partially* deranged. The flippancy of the anti-phrenologists has always appeared to us in exact proportion to their ignorance of the doctrines which they oppugn.

---

